# A New Anti-epileptic and Nervine

**Published:** 1886-01-20

**Authors:** 


**A New Anti-epileptic and Nervine,** (St. Louis Medical Journal) said by the Spanish and South American Medical journals to be of great value, is the fruit of a species of caper, the *capparis corriacea*, a native of Peru. It is used in the shape of an infusion, 3 drams of the powdered fruit infused in good red wine being a dose. It undoubtedly possesses considerable sedative power and is valuable in hysteria and similar nervous disorders, and is relied upon by native physicians as a powerful agent in preventing epileptic attacks.

